# Novel *KLK4* Mutations Cause Hypomaturation Amelogenesis Imperfecta

**DOI:** 10.3390/jpm12020150

**Published:** 2022-01-24

**Authors:** Yejin Lee, Hong Zhang, Figen Seymen, Youn Jung Kim, Yelda Kasimoglu, Mine Koruyucu, James P. Simmer, Jan C.-C. Hu, Jung-Wook Kim

**Affiliations:** 1 Department of Pediatric Dentistry, School of Dentistry & DRI, Seoul National University, Seoul 03080, Korea; lyj72255621@gmail.com (Y.L.); ykim71@snu.ac.kr (Y.J.K.); 2 Department of Biologic and Materials Sciences & Prosthodontics, School of Dentistry, University of Michigan, Ann Arbor, MI 48109, USA; zhanghon@umich.edu (H.Z.); jsimmer@umich.edu (J.P.S.); janhu@umich.edu (J.C.-C.H.); 3 Department of Pedodontics, Faculty of Dentistry, Istanbul University, Istanbul 34116, Turkey; fseymen@istanbul.edu.tr (F.S.); yeldakasimoglu@gmail.com (Y.K.); mine.yildirim@istanbul.edu.tr (M.K.); 4 Department of Molecular Genetics, School of Dentistry & DRI, Seoul National University, Seoul 03080, Korea

**Keywords:** whole exome sequencing, kallikrein 4, amelogenesis imperfecta, genetic diseases, hypomaturation, zymography

## Abstract

Amelogenesis imperfecta (AI) is a group of rare genetic diseases affecting the tooth enamel. AI is characterized by an inadequate quantity and/or quality of tooth enamel and can be divided into three major categories: hypoplastic, hypocalcified and hypomaturation types. Even though there are some overlapping phenotypes, hypomaturation AI enamel typically has a yellow to brown discoloration with a dull appearance but a normal thickness indicating a less mineralized enamel matrix. In this study, we recruited four Turkish families with hypomaturation AI and performed mutational analysis using whole exome sequencing. These analyses revealed two novel homozygous mutations in the *KLK4* gene: a nonsense mutation in exon 3 (NM_004917.4:c.170C>A, p.(Ser57*)) was found in families 1, 2 and 3 and a missense mutation in exon 6 (c.637T>C, p.(Cys213Arg)) in family 4. Functional analysis showed that the missense mutation transcript could not translate the mutant protein efficiently or generated an unstable protein that lacked functional activity. The two novel inactivating *KLK4* mutations we identified caused a hypomaturation AI phenotype similar to those caused by the four previously described *KLK4* nonsense and frameshift mutations. This study improves our understanding of the normal and pathologic mechanisms of enamel formation.

## 1. Introduction

Tooth enamel, the hardest tissue in the human body, is the outermost covering of the crown of the teeth. Healthy enamel has a lustrous light yellow to bluish white color. To achieve an exceptional hardness and beautiful shape, the enamel-forming process (amelogenesis) is under tight control and strict surveillance from start to finish [1]. Therefore, minor alterations that might be tolerated during the development of other tissues, result in tooth enamel malformations of a localized or generalized form. Non-inheritable enamel defects are caused by environmental factors, such as nutritional deficiency, excessive chemical elements, high fever and selected medical conditions [2].

Amelogenesis imperfecta (AI) is a collection of rare genetic disorders affecting the tooth enamel [3]. Therefore, AI is heterogeneous in genetic etiology and in clinical phenotypes. Amelogenesis occurs in discrete but continuous processes: secretion, calcification and maturation [4]. The ameloblasts that differentiate through a series of ectodermal-mesenchymal interactions begin to secret enamel matrix proteins onto an accumulated, collagen-rich predentin matrix secreted by odontoblasts [5]. The secreted enamel matrix calcifies to form thin but long enamel crystallites on the surface of recently mineralized dentin. During the maturation stage, the enamel crystallites become thicker and wider and interlock. A disruption in any step of amelogenesis causes a process-specific form of AI (hypoplastic, hypocalcified and hypomaturation), but sometimes the phenotype can be a mixed type [6].

Hypoplastic AI is characterized by a thin but hard enamel caused by an insufficient thickness (or volume) of formed enamel. The phenotype is diverse: from localized enamel pits and grooves to a generalized hypoplastic enamel with a smooth or rough surface and even to enamel agenesis in the most severe cases. Hypocalcified AI is characterized by a cheesy soft enamel but with a normal thickness, which easily breaks down following tooth eruption and results in a rough and discolored surface. Hypomaturation AI is characterized by a less-mineralized weak enamel with a normal thickness. Increased residual organic components in the enamel result in a dull and dark yellow to brown discoloration. Weak enamel tends to fracture off or break down with attrition [7].

To date, mutations in genes encoding ODAPH (odontogenesis associated phosphoprotein; OMIM *614829) [8], MMP20 (matrix metalloproteinase 20, OMIM *604629) [9], KLK4 (kallikrein related peptidase 4, OMIM *603767) [10], WDR72 (wd repeat-containing protein 72, OMIM *613214) [11], SLC24A4 (solute carrier family 24 member 4, OMIM *609840) [12] and GPR68 (G protein-coupled receptor 68, OMIM *601404) [13] have been involved in hypomaturation AI in an autosomal recessive inheritance pattern. Mutations in the *DLX3* (distal-less homeobox 3, OMIM *600525) gene [14,15] and specific *AMELX* (amelogenin, OMIM *300391) mutations [16,17] cause a mixed hypoplastic and hypomaturation form of AI that are inherited in an autosomal dominant and X-linked pattern, respectively.

In this study, we investigated four Turkish families with a hypomaturation AI phenotype and performed whole exome sequencing with selected family members. Mutational analyses revealed novel homozygous mutations (nonsense and missense mutations) in the *KLK4* gene, one of the enamel matrix proteinases, and functional analysis confirmed the mutational effect. This report expands the mutational spectrum of the *KLK4* gene causing enamel hypomaturation and advances our understanding of normal and pathologic amelogenesis.

## 2. Materials and Methods

### 2.1. Enrollment of Study Subjects

The study protocol was independently reviewed and approved by the institutional review boards of Seoul National University Dental Hospital (CRI05003G and 10 December 2020), Istanbul University (No: 2008/931 and 20 September 2019) and the University of Michigan (H03-00001835-M1 and 6 May 2021). Informed consent was obtained from all subjects involved in this study with understanding of the research-related information. Clinical and radiographic examinations were performed, and saliva samples collected.

### 2.2. DNA Isolation and Whole Exome Sequencing

Genomic DNA was isolated from the saliva samples, and its quality and quantity determined. DNA samples from selected individuals in each family (Table S1) were submitted to Theragen Bio (Family 1; Seongnam-si, Korea), BGI (Family 2; Shenzhen, China) and Johns Hopkins University Center for Inherited Disease Research (Family 3 and 4; CIDR, Baltimore, MD, USA) for exome capturing and generation of paired-end sequencing reads.

### 2.3. Analysis of the Sequencing Reads

The paired-end sequence reads were aligned to the reference human genome assembly (hg37) using the Burrows-Wheeler Aligner [18]. A series of bioinformatics analysis procedures using Samtools and the Genome Analysis Tool Kit [19,20] was performed to get a list of sequence variants including single nucleotide and small indel (insertion and deletion) variations. Annotation was performed using Annovar [21] with dbSNP build 147, and a minor allele frequency (MAF) of 0.01 was applied as a cutoff value to filter the variants.

### 2.4. Polymerase Chain Reaction (PCR) and Sanger Sequencing

Potentially pathogenic sequence variations and their segregations among family members were confirmed via Sanger sequencing using the following PCR primers: *KLK4* exon 3 for families 1 to 3 (sense 5′-GCCCCCAGCCCTGACTCG-3′ and antisense 5′-TCACGCACTGCAGCACGGTA-3′) and *KLK4* exon 6 for family 4 (sense 5′-GGGATCTGGAATGGGACTT-3′ and antisense 5′-GGGGATCTGTACCCTTGG-3′). Sanger sequencing was performed for all participating family members at Macrogen (Seoul, Korea) or Eurofins Genomics (Louisville, KY, USA).

### 2.5. PCR Mutagenesis

The mammalian expression vector pcDNA3.1 (Thermo Fisher Scientific, Waltham, MA, USA) expressing human KLK4 [22] was used for the mutagenesis. We introduced the *KLK4* mutation identified in patients (NM_004917.4:c.637T>C, p.(Cys213Arg)) using PCR mutagenesis (sense: 5′-GCCCCTGATCCGCAACGGGTAC-3′, antisense: 5′-GTACCCGTTGCGGATCAGGGGC-3′). The sequence of the mutated pcDNA3.1-*KLK4* vector was confirmed via direct plasmid sequencing.

### 2.6. Western Blotting

HEK293T cells in a 100 mm culture dish were transiently transfected with 5 µg of the wild type or mutant pcDNA3.1 plasmid vectors using 15 µL of GenJet^TM^ In Vitro DNA Transfection Reagent (Ver II) (SignaGen, Frederick, MD, USA). After 6 h, the cells were washed twice with phosphate-buffered saline (PBS) and incubated in DMEM medium without FBS for 48 h. After 48 h of incubation, the cells were harvested with RIPA lysis buffer including protease inhibitors. Then, 4 mL of conditioned medium from the cell culture was collected and concentrated to 200 µL using an Amicon ultra-4 centrifugal filter unit (Merck Millipore, Burlington, MA, USA). Cell lysate (25 µg) and concentrated media (wild type to mutant loading ratio was 1:10) were separated using 12% SDS-PAGE gel and transferred to a PVDF membrane (Merck Millipore). The membrane was blocked for 2 h at room temperature with 5% skim milk in 1× TBS-T and incubated overnight at 4 °C with rabbit polyclonal anti-Kallikrein 4 antibody (Abcam, Cambridge, UK) diluted in 5% skim milk buffer (1:10,000). After incubation with the primary antibody, the membrane was washed 3 times with 1× TBS-T for 10 minutes. After washing, the membrane was incubated for 2 h at room temperature with diluted (1:10,000) goat anti-Rabbit IgG secondary antibody (Thermo Fisher Scientific).

### 2.7. Zymography

The activities of the expressed wild type and mutant KLK4 proteins were assayed using gelatin zymography. Thermolysin (Sigma-Aldrich, St. Louis, MO, USA) was dissolved in a buffer containing 50 mM Tris and 5 mM CaCl_2_ at a concentration of 20 µg/mL. Then, 10 µL of concentrated media was mixed with 1 µL of thermolysin solution, and the mixture was incubated at 37 °C for 16 h. Samples were mixed with 5× non-reducing loading buffer and electrophoresed on 12% SDS-PAGE gel containing 0.1% gelatin (Sigma-Aldrich) at 80 V for 5 h in an ice box. After electrophoresis, the gel was incubated in 1× renaturing buffer (Novex, Waltham, MA, USA) at room temperature for 15 min and this step was repeated 3 times. After the renaturing step, the gel was incubated at 37 °C for 24 h in 1× developing buffer (Novex). The zymogram was stained with 0.5% Coomassie brilliant blue R-250 (Amresco, Radnor, PA, USA), dissolved in 45% MeOH, 10% acetic acid staining solution for 1 h and finally visualized after washing with destaining solution (25% EtOH, 10% acetic acid).

## 3. Results

### 3.1. Family 1~3

**Figure 1:**
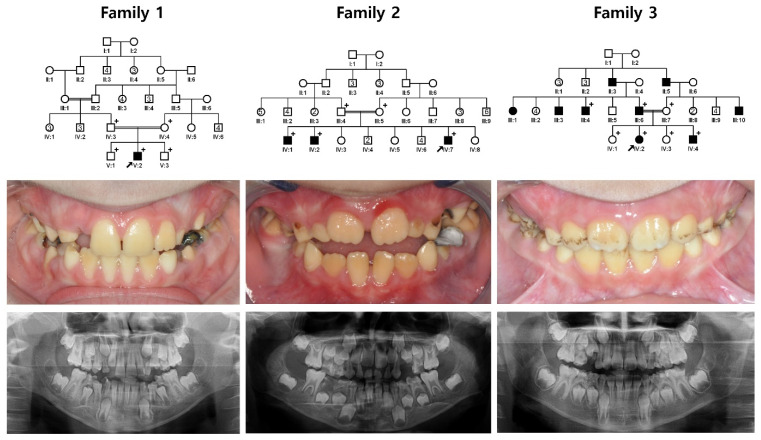
Family pedigrees, frontal clinical photos and panoramic radiographs. Clinical images and panoramic radiographs show generalized hypomaturation AI with yellow-brown discoloration. Some teeth of the proband in family 3 had external staining. Plus (+) symbols indicate individuals who participated in this study, and a black arrow identifies the proband in each family. Numbers in the symbol indicate the number of siblings.

The proband of family 1 was a 10-year-old second child from a consanguineous marriage who exhibited a generalized dark-yellow discoloration of the dentition without other systemic illnesses (Figure 1 and Figure S1). There were no other affected individuals in the family. The proband of family 2 was a 7-year-old eleventh child from a consanguineous marriage. His two older brothers were also affected with the same dental phenotype. The proband had been treated for acute lymphocytic leukemia, but there was no remarkable past medical history in the other affected siblings. The proband of family 3 was a 9-year-old girl from a consanguineous family. Her dentition also had a generalized dark yellow to brown discoloration in addition to extrinsic dark-brown staining. The father was also similarly affected, and interestingly, there were several affected individuals in the pedigree.

Mutational analysis revealed a novel homozygous nonsense mutation in the *KLK4* gene. The mutation was a transversional substitution of a cytosine to an adenine at cDNA position 170 in exon 3 (NM_004917.4:c.170C>A) (Figures S2–S4). This mutation would change the amino acid serine (encoded by the nucleotides TCG) to an amber stop codon (encoded by the nucleotides TAG) at amino acid position 57 (p.(Ser57*)). Because this nonsense mutation generates a premature termination codon in exon 3 of the *KLK4* gene (one 5′ non-coding and five coding exons), the mutant mRNA transcript would likely be degraded by the nonsense-mediated decay system instead of synthesizing a truncated protein [23,24]. Therefore, the mutational effect of this mutation would be a loss of KLK4 function during amelogenesis. This mutation was listed in the dbSNP build 151 with accession number rs1185328501 and was reported in the Genome Aggregation Database (gnomAD: https://gnomad.broadinstitute.org/, accessed on 13 December 2021) with an allele count of 1 (in non-Finnish European population) out of a total allele number of 251416. All participating affected individuals in families 1, 2 and 3 had the same homozygous mutation, and it turned out that they shared the same disease allele. Therefore, it seems that they inherited the mutation from a common founder in the Turkish population.

### 3.2. Family 4

**Figure 2:**
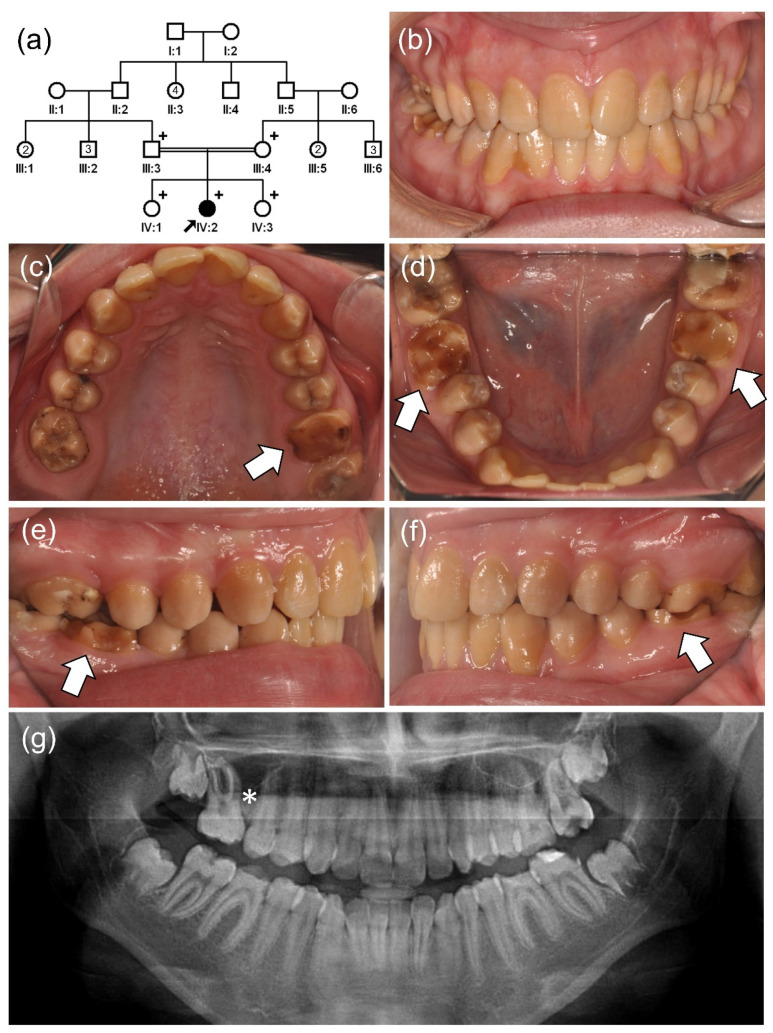
(**a**) Pedigree of family 4. The black arrow denotes the proband. Plus (+) symbols indicate participating individuals in this study. Number in the symbol indicates the number of siblings. Consanguineous marriage is shown by a double line. (**b**–**f**) Clinical photos of the proband (IV:2). Hypomatured enamel exhibits a generalized yellow-brown discoloration. Early loss of weak enamel can be seen in the permanent first molars. (**g**) Panoramic radiograph shows hypomineralized enamel with reduced radiodensity. The maxillary right first molar (*) was extracted due to severe destruction.

The proband of family 4 was a 15-year-old second girl from a consanguineous marriage (Figure 2). The pregnancy and delivery of the proband were uneventful, and there was no remarkable medical history. However, her dentition had a generalized brown discoloration and hypomatured enamel. Her first molars looked to be worn down by attrition, and the maxillary right first molar was extracted due to severe destruction.

**Figure 3:**
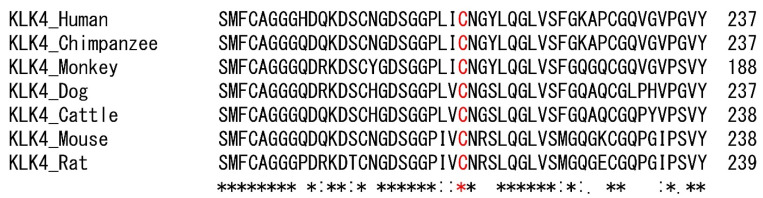
Sequence alignment of vertebrate orthologs. The asterisk indicates complete conservation.

Mutational analysis revealed a homozygous missense mutation in the *KLK4* gene. The mutation was a transitional substitution of a thymine to a cytosine at cDNA position 637 in exon 6, the last exon (NM_004917.4:c.637T>C) (Figure S5). This mutation would change the amino acid cysteine (encoded by nucleotides TGC) to an arginine (encoded by nucleotides CGC) at amino acid position 213 [p.(Cys213Arg)] and therefore prevent formation of a Cys213-Cys148 disulfide bond. The cysteine at this position is completely conserved among vertebrate homologs (Figure 3), and in silico programs predicted it as a harmful variation: Sift predicted it as deleterious with a score of 0 [25]; PolyPhen-2 predicted it to be probably damaging with a score of 1.000 (sensitivity: 0.00; specificity: 1.00) [26], and the Combined Annotation Dependent Depletion (CADD) score was also very high (25.5) [27]. The mutation was listed in the dbSNP build 151 with accession number rs1266288524 and reported in the gnomAD database with an allele count of 2 (in a non-Finnish European population) out of a total allele number of 251,326.

**Figure 4:**
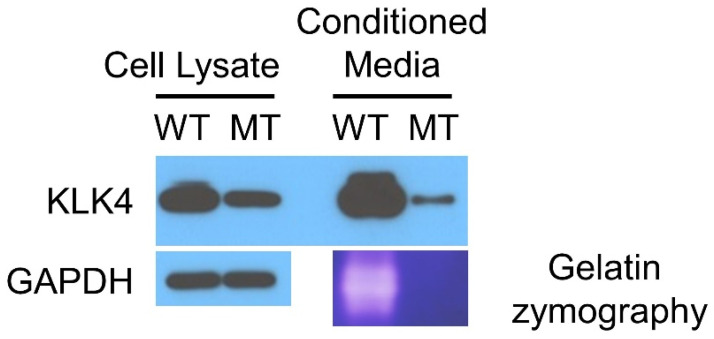
Western blot of the cell lysate and conditioned media. The names of the antibodies used are listed on the left. Gelatin zymography showed the loss of proteolytic function for mutant KLK4. WT, wild type sample transfected with normal *KLK4* pcDNA3.1 vector; MT, mutant sample transfected with mutant *KLK4* pcDNA3.1 vector.

Western blotting revealed that the mutant KLK4 can be produced but in a reduced amount, and its secretion is further reduced (Figure 4). The zymography study also revealed no functional activity of the mutant KLK4. It seems that the missense mutation causes misfolding of the mutant protein, and the misfolded protein could not be produced and secreted efficiently. The mutant KLK4 was secreted in a greatly reduced amount and was functionally inactive. Therefore, the mutational effect of this missense mutation would be also a loss of KLK4 function in amelogenesis (Table 1).

## 4. Discussion

Kallikrein enzymes are serine proteases that are composed of plasma kallikrein (KLKB1, OMIM *229000) and kallikrein related peptidases (KLKs) [30]. The KLK family locates at chromosomal location 19q13.3-q13.4, in a cluster of a 15 functional *KLK* gene family that is 265 kb long and not interrupted by non-*KLK* genes [31]. A phylogenetic study suggested that the *KLK4* gene arose via gene duplication from the *KLK5* gene [32]. The *KLK4* gene locates between the *KLK2* and *KLK5* genes in the cluster. The KLKs share sequence similarity ranges from about 40 to 80% and similar gene structures: they have a variable number of 5′ non-coding exons (0~2) but have five coding exons for encoding the mature proteins of approximately 230 amino acid residues in length [33].

All of the KLKs are initially synthesized as preproenzymes and then become proenzymes with the removal of a signal peptide [34]. Removal of a short activation peptide via proteolysis results in a mature active chymotrypsin-like serine proteinase. Structurally, there is a catalytic triad of amino acids that are completely conserved in all KLKs: histidine, aspartic acid and serine [35]. The number (10–12) and position of the cysteine residues are also highly conserved and are expected to form disulfide bridges to provide proper folding and stability [36].

Human *KLK4* has one 5′ non-coding exon and five coding exons to encode a preproenzyme of 254 amino acids. With the removal of the signal peptide (26 amino acids), the proenzyme is secreted into the enamel matrix from the transitional to maturation stages. Another important enamel matrix proteinase, MMP20, is expressed from the secretory to transitional stages and is believed to process enamel matrix structural proteins, such as AMELX, ENAM and AMBN, into smaller functional units [37]. Secreted KLK4 should be activated with the removal of a 4-amino-acid activation peptide (SCSQ) at the N-terminus [38]. MMP20 can activate KLK4 and then is deactivated by mature KLK4 [39]. However, there seems to be another activator in the enamel matrix because KLK4 is still active in *Mmp20* knockout mice [40]. Because there is no KLK4 activating enzyme in the HEK293T cell media, we used thermolysin, a thermostable metalloproteinase from Gram-positive bacteria, which was previously shown to activate KLK4 in vitro [39].

The mature KLK4 of 234 amino acids is a glycosylated chymotrypsin-like serine protease. Human KLK4 has a single potential N-glycosylation site (predicted to be Asn169), in contrast to the three N-glycosylation sites in the mouse and pig KLK4 [41]. Glycosylation is likely important for protein stability by protecting against proteolytic degradation [42]. The locations of the highly conserved catalytic triad are His71, Asp116 and Ser207 [43]. The missense mutation identified in this study was p.(Cys213Arg), changing a highly conserved cysteine to an arginine. Active KLK4 has six disulfide bridges to provide proper folding, and indeed, the mature KLK4 has exactly 12 cysteine residues (Cys37-Cys167, Cys56-Cys72, Cys141-Cys241, Cys148-Cys213, Cys178-Cys192 and Cys203-Cys228) [38]. The mutant protein would be misfolded by disrupting an essential structural interaction. Therefore, the intracellular protein production is affected and reduced. Furthermore, zymography showed that the mutant protein lacked functional activity.

In summary, we identified two novel *KLK4* mutations in four Turkish families with hypomaturation AI. The nonsense mutation with a premature termination codon was predicted to be degraded by the nonsense-mediated decay system, and the missense mutation was demonstrated to be generated and exported to the enamel matrix in a reduced amount but lacked functional activity. This study expands the mutational spectrum of the *KLK4* gene causing hypomaturation AI and advances our understanding of enamel maturation. Further studies to find a way to increase the degree of enamel maturation could be useful for AI patients and the general population as well.

## Figures and Tables

**Table 1:** Disease-causing mutations in *KLK4* gene.

Location	cDNA	Protein	Mode of Inheritance	References
Exon 3	c.170C>A	p.(Ser57*)	AR homo	This report
Exon 4	c.245delG	p.(Gly82Alafs*87)	AR homo	Wang et al. (2013) [28]
Exon 4	c.458G>A	p.(Trp153*)	AR homo	Hart et al. (2004) [10]
Exon 6	c.620_621delCT	p.(Ser207Trpfs*38)	AR homo	Seymen et al. (2015) [22]
Exon 6	c.632delT	p.(Leu211Argfs*37)	AR homo	Smith et al. (2017) [29]
Exon 6	c.637T>C	p.(Cys213Arg)	AR homo	This report

Sequences based on the reference sequence for mRNA (NM_004917.4) and protein (NP_004908.4), where the A of the ATG translation initiation codon is nucleotide 1.

## Data Availability

The data presented in this study are openly available in ClinVar (http://www.ncbi.nlm.nih.gov/clinvar, accessed on 16 December 2021), Submission ID: SCV002032321 and SCV002032322.

## Supplementary Materials

The following are available online at https://www.mdpi.com/article/10.3390/jpm12020150/s1, Figure S1: Clinical photos of the probands in families 1~3, Figure S2: Sequencing chromatograms of the participating individuals of family 1, Figure S3: Sequencing chromatograms of the participating individuals of family 2, Figure S4: Sequencing chromatograms of the participating individuals of family 3, Figure S5: Sequencing chromatograms of the participating individuals of family 4, Table S1: Statistics for exome sequencing.
