# Supplementary material for: Novel KLK4 Mutations Cause Hypomaturation Amelogenesis Imperfecta
Source: J Pers Med. 2022 Jan 24;12(2):150. doi: 10.3390/jpm12020150 (PMC8878363; doi:10.3390/jpm12020150)
Supplement: Supplementary file 1 [file jpm-12-00150-s001.zip › jpm-1536606-supplementary.pdf]

Novel *KLK4* Mutations Cause Hypomaturation Amelogenesis Imperfecta

Yejin Lee [1,†], Hong Zhang [2,†], Figen Seymen [3], Yelda Kasimoglu [3], Mine Koruyucu [3], James P. Simmer [2], Jan C.-C. Hu [2] and Jung-Wook Kim[1,4,*]

[1]Department of Pediatric Dentistry,
School of Dentistry & DRI, Seoul National University, Seoul 03080, Korea.
[2]Department of Biologic and Materials Sciences & Prosthodontics, School of Dentistry, University of Michigan, Ann Arbor, MI 48109, USA.
[3]Department of Pedodontics, Faculty of Dentistry, Istanbul University, Istanbul 34116, Turkey
[4]Department of Molecular Genetics,
School of Dentistry & DRI, Seoul National University, Seoul 03080, Korea.

† These authors equally contributed to this work.
* Correspondence: pedoman@snu.ac.kr (J.-W.K.)

Table S1. Statistics for exome sequencing.

Sample		Total reads	Mapping rate (%)	Median target coverage	Coverage of target region (%)	Fraction of target covered with at least	
						20X	10X
Family 1	V:2	74,485,830	98.2	70	96.4	91.6	94.9
Family 2	III:4	123,184,317	99.6	92	96.2	92.9	94.9
	III:5	126,406,940	99.9	95	96.1	93.3	95.0
	IV:2	89,232,516	99.6	66	96.2	90.4	94.3
Family 3	III:6	198,709,274	99.7	120	99.5	97.8	98.9
	IV:1	125,774,962	99.4	73	99.4	95.7	98.4
Family 4	III:3	99,282,916	99.4	63	99.4	94.6	98.1
	III:4	136,200,546	98.9	74	99.4	96.4	98.5
	IV:2	142,866,102	99.2	80	99.3	96.6	98.5

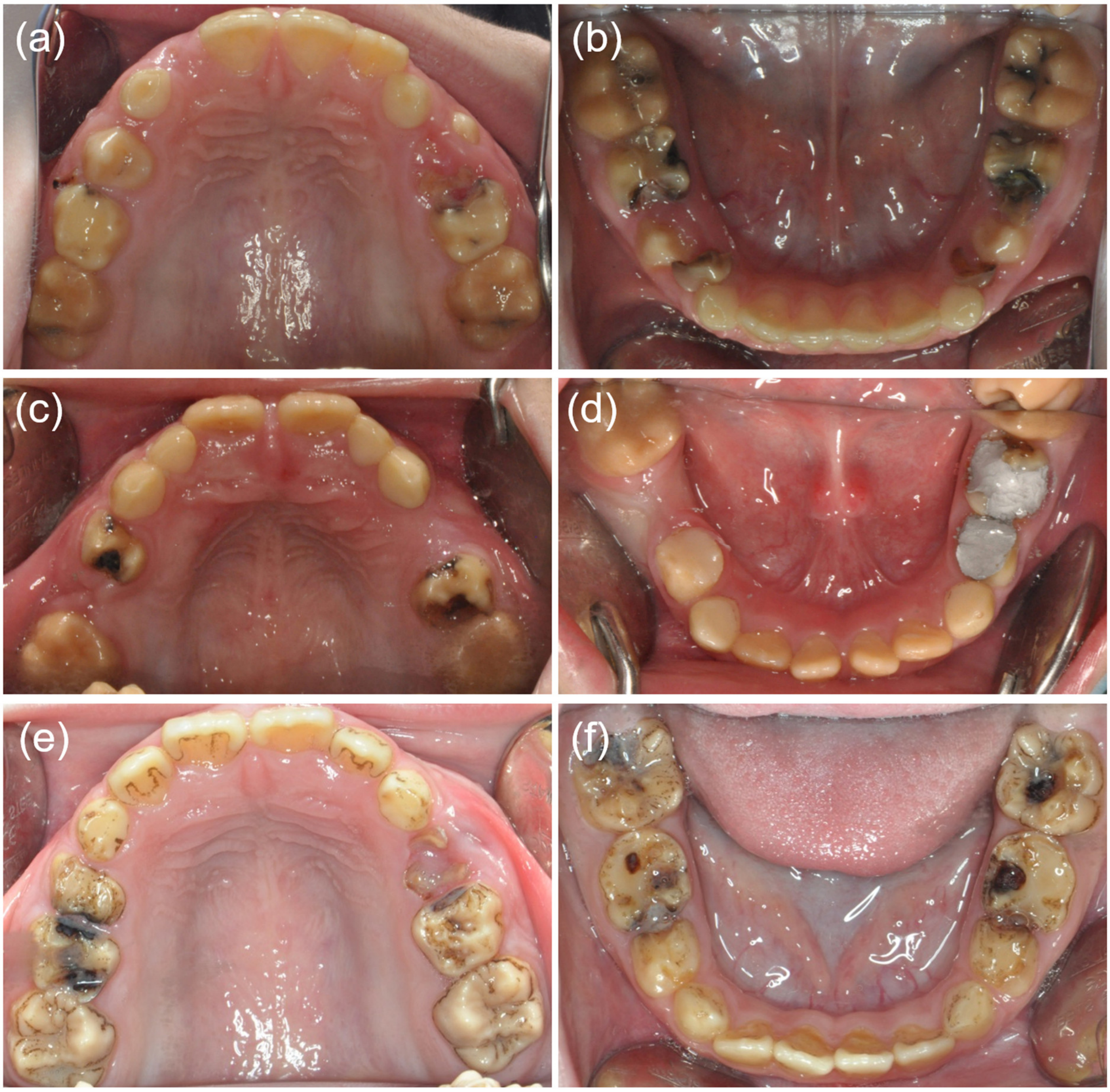

Figure S1. (**a,b**) Clinical photos of the proband in family 1. (**c,d**) Clinical photos of the proband in family 2. (**e,f**) Clinical photos of the proband in family 3.

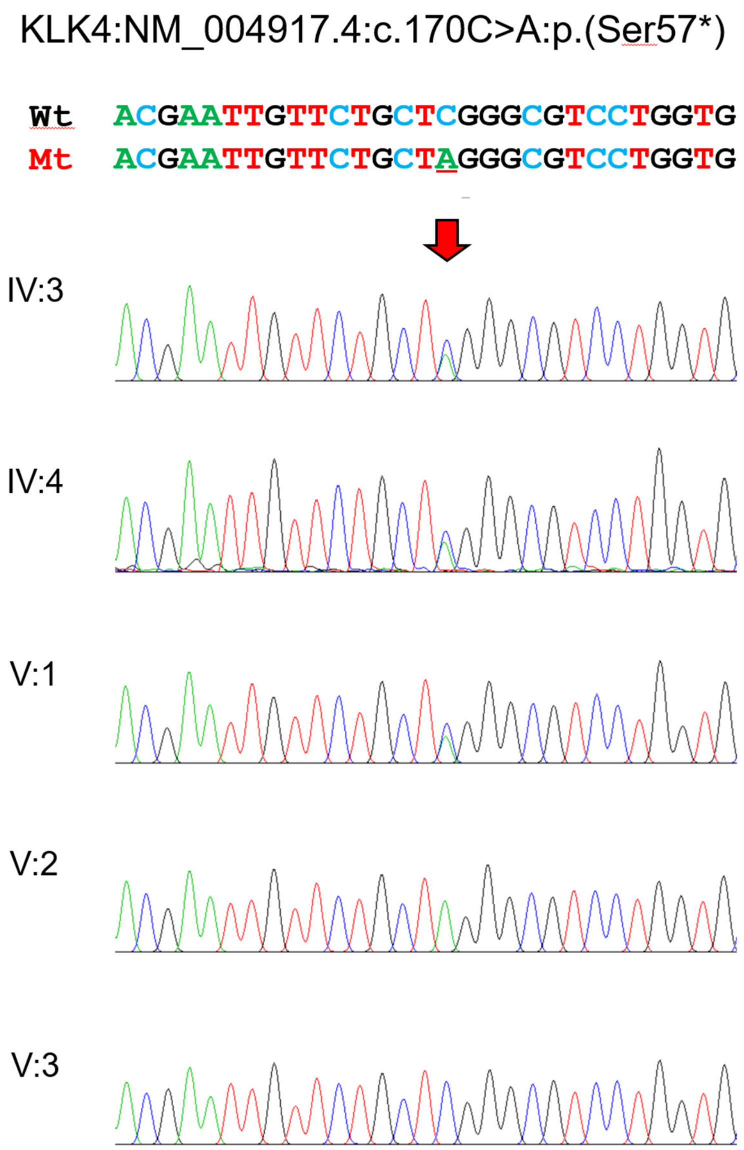

Figure S2. Sequencing chromatograms of the participating individuals of family 1. Wild type (Wt) and mutant (Mt) nucleotide sequences are shown above the chromatograms. Nucleotides affected by the mutation are underlined. The location of the mutation is indicated with a red arrow. Individual identifications are indicated on the left side of each chromatogram.

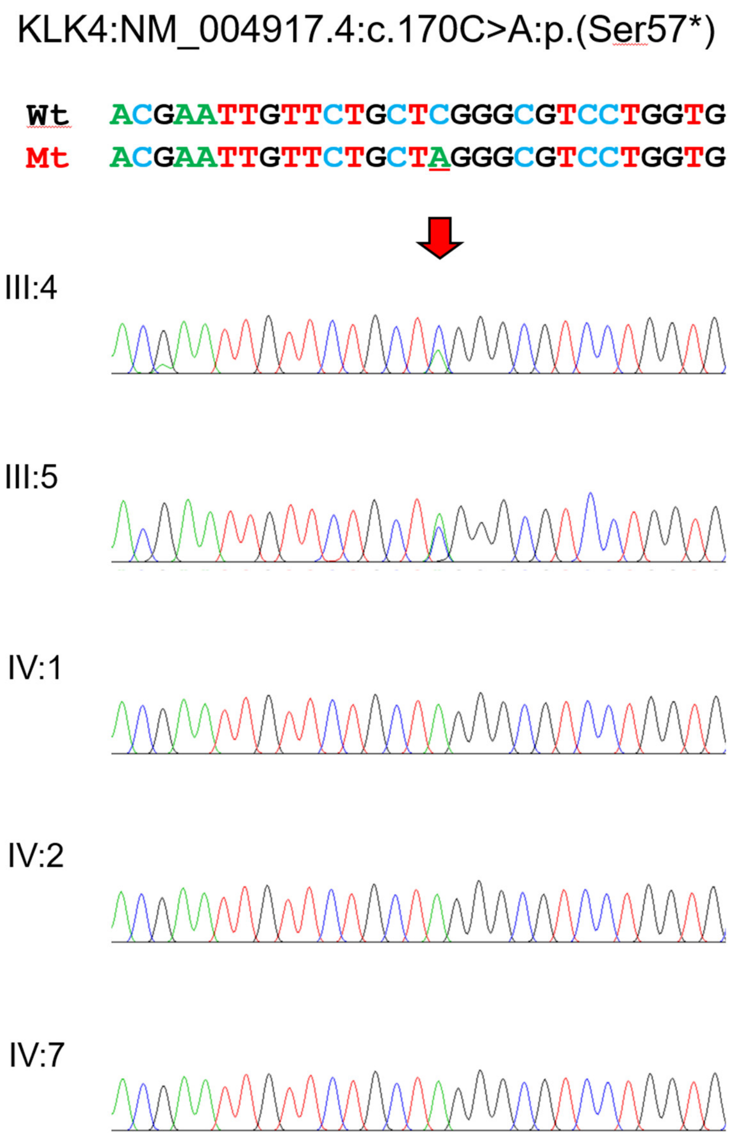

Figure S3. Sequencing chromatograms of the participating individuals of family 2. Wild type (Wt) and mutant (Mt) nucleotide sequences are shown above the chromatograms. Mutated nucleotide is underlined. The location of the mutation is indicated with a red arrow. Individual identifications are indicated on the left side of each chromatogram.

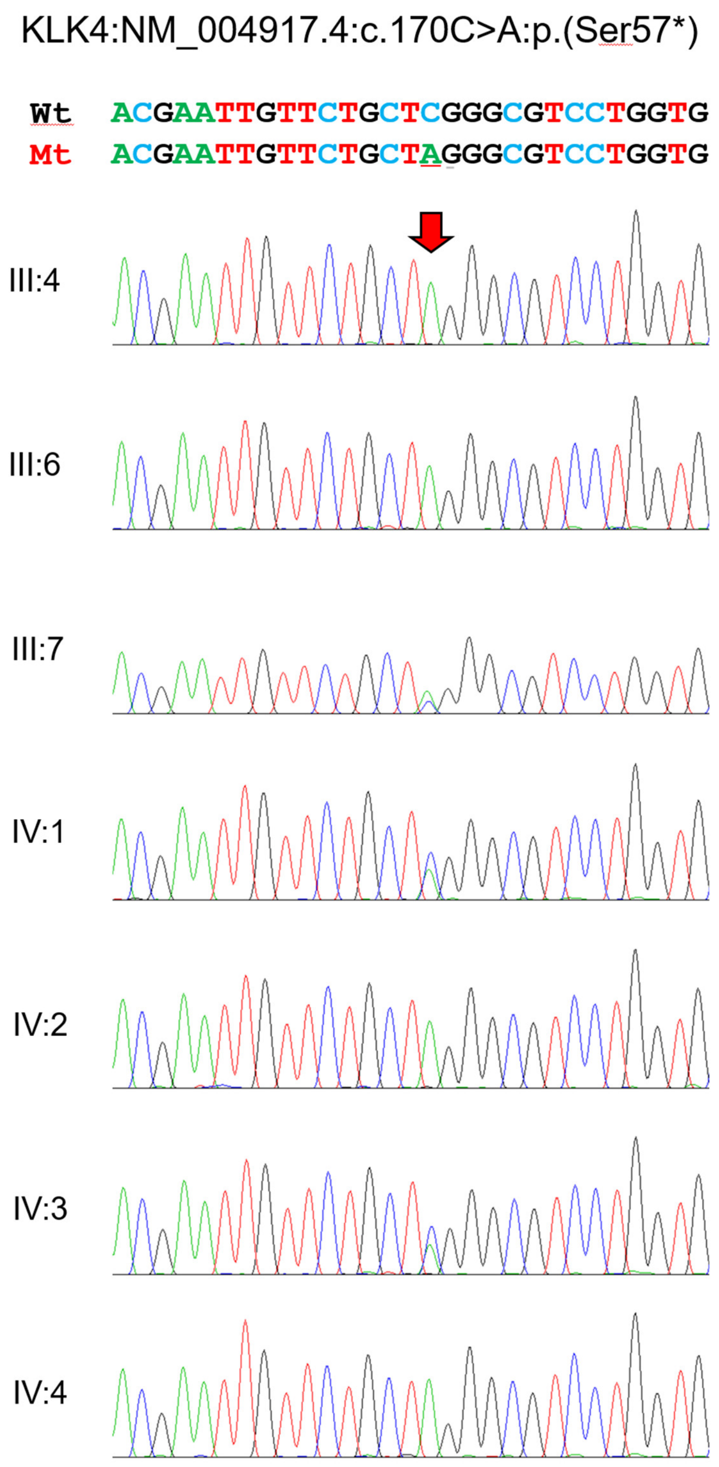

Figure S4. Sequencing chromatograms of the participating individuals of family 3. Wild type (Wt) and mutant (Mt) nucleotide sequences are shown above chromatograms. Mutated nucleotide is underlined. The location of the mutation is indicated with a red arrow. Individual identifications are indicated on the left side of each chromatogram.

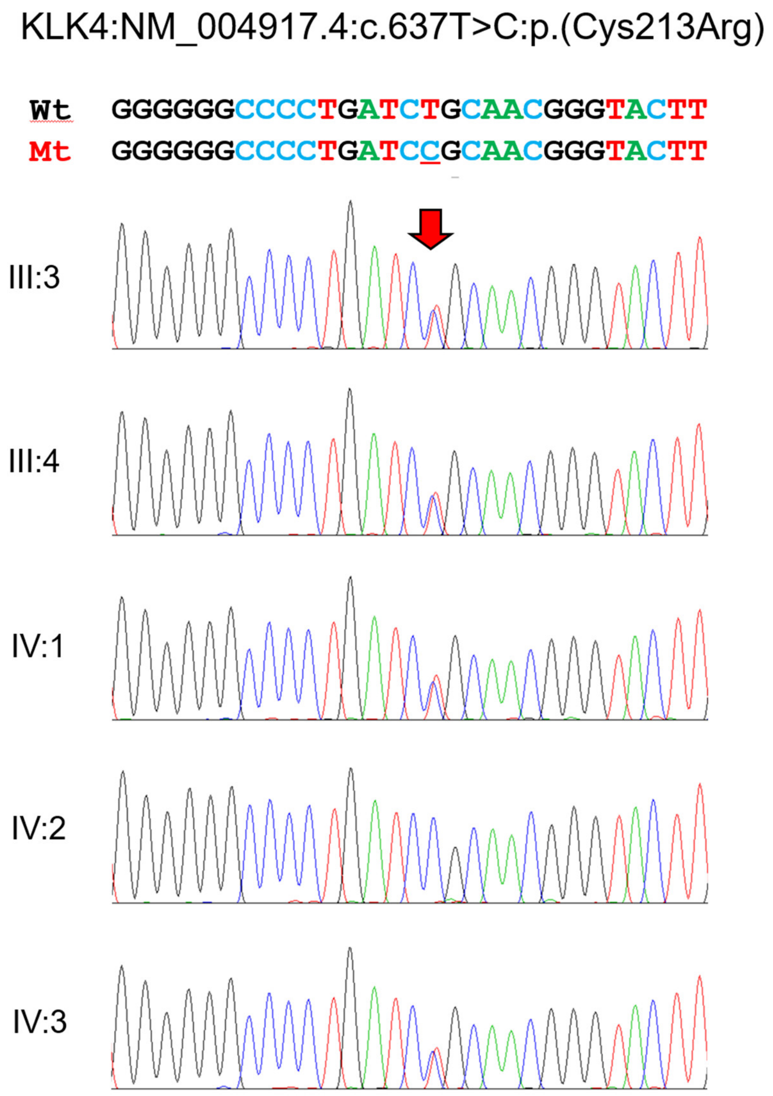

Figure S5. Sequencing chromatograms of the participating individuals of family 4. Wild type (Wt) and mutant (Mt) nucleotide sequences are shown above chromatograms. Mutated nucleotide is underlined. The location of the mutation is indicated with a red arrow. Individual identifications are indicated on the left side of each chromatogram.
